# Can Vitamin D Reduce Inflammation? The Influence of Supplementation on Selected Immunological Markers

**DOI:** 10.3390/ijms25147592

**Published:** 2024-07-11

**Authors:** Martyna Lis, Natalia Niedziela, Jowita Adamczyk-Zostawa, Jolanta Zalejska-Fiolka, Michał Błachut, Jarosław Szczygieł, Agata Świętek, Monika Adamczyk-Sowa

**Affiliations:** 1 Department of Neurology, Faculty of Medical Sciences in Zabrze, Medical University of Silesia, 40-055 Katowice, Poland; martyna.anna.nowak@gmail.com (M.L.); m.adamczyk.sowa@gmail.com (M.A.-S.); 2 Department of Ophthalmology, Faculty of Medical Sciences in Zabrze, Medical University of Silesia, 40-055 Katowice, Poland; 3 Department of Biochemistry, Faculty of Medical Sciences in Zabrze, Medical University of Silesia, 40-055 Katowice, Poland; 4 Department of Psychiatry, Faculty of Medical Sciences in Zabrze, Medical University of Silesia, 40-055 Katowice, Poland; 5 Silesia LabMed Research and Implementation Center, Medical University of Silesia in Katowice, 19 Jordana St., 41-808 Zabrze, Poland; agata.swietek@sum.edu.pl

**Keywords:** multiple sclerosis, inflammation, immune system, vitamin D, supplementation

## Abstract

There is increasing evidence that vitamin D (VitD) supplementation may reduce inflammation in individuals with multiple sclerosis (MS). The aim of this study was to evaluate the effect of different doses of VitD on selected markers of inflammation in patients with relapsing–remitting MS (RRMS). Participants were divided depending on the supplemented dose of VitD into a high-dose (2000 IU/d; HD) group and a low-dose (15,960 IU/month; LD) group (*n* = 23 and *n* = 29, respectively). The concentration of 25(OH)D and the levels of CXCL16, PTX3, ALCAM, IL-1RA, and OPG were measured initially and after six months of VitD supplementation in blood serum. A significant increase in the concentrations of CXCL16, PTX3, and OPG was observed during the study (*p* = 0.02, *p* = 0.01, and *p* < 0.01, respectively). Furthermore, a higher increase in PTX3 and OPG in the LD group was observed (*p* = 0.04 and *p* = 0.03, respectively). A significant positive correlation was observed between the 25(OH)D serum concentration and PTX3 (R = 0.28, *p* < 0.05) and OPG (R = 0.28, *p* < 0.05) only at the beginning of the study. In patients with RRMS, such doses of VitD might be too low to induce obvious beneficial effects on the pro-inflammatory and inflammatory balance.

## 1. Introduction

Multiple sclerosis (MS) is a complex neurodegenerative disease associated with chronic inflammation, oxidative stress (OS), and blood–brain barrier (BBB) disruption, leading to neuronal and axonal damage and demyelination [1]. The disease mainly affects young adults and is a common cause of disability, resulting in a multitude of neurological impairments [2].

The most common form is relapsing–remitting MS (RRMS), in which relapses are caused by inflammation due to immunological dysfunction in the central nervous system (CNS) [3]. Different inflammatory processes are the main pathological drivers of the disease. The autoimmune reaction of activated T lymphocytes leads to the upregulation of pro-inflammatory factors and the activation of microglia/macrophages, leading to pathological changes underlying MS. In addition, the above-mentioned oxidative injury, through the generation of reactive oxygen and nitrogen species (ROS, RNS), creates an autotoxic feedback loop and intensifies existing inflammation [4,5].

Furthermore, scientists assume that MS is caused by the interplay of environmental and genetic risk factors [6]. Due to the frequent prevalence of the disease at higher latitudes, hypovitaminosis D is suspected to play a role in MS [7]. In addition to the classic role of vitamin D (VitD) in calcium and phosphate homeostasis, it also plays a role in cell proliferation and differentiation and participates in the responses of the immune and nervous systems. It acts as an immunomodulator, regulating the secretion of different cytokines and shifting the immune response towards a less inflammatory profile. Additionally, it promotes the expression of regulatory T lymphocytes (Tregs) and growth factors, influences cell signaling, and has a response to OS (antioxidant effect), thus contributing to the protection of the CNS [8,9,10,11]. The issue of the impact of VitD supplementation on selected markers of a pro- and antioxidant balance has been discussed in our previous research.

VitD deficiency is prevalent in the general population, affecting over 1 billion people worldwide [12], and it is more pronounced in patients with MS than in healthy controls [13]. VitD status may not only be related to the susceptibility to MS in genetically predisposed individuals, but it may also influence the activity of the disease. It has been observed that patients with a low serum concentration have higher disease activity [14]. Previous research has shown that reduced VitD levels may increase the likelihood of new MS relapses, enable the detection of new plaques in magnetic resonance imaging (MRI), and can be associated with a higher degree of disability compared to MS patients with higher 25(OH)D levels [15]. There is increasing evidence that supplemental VitD may exert immunological effects in MS individuals associated with reducing underlying immune and inflammatory processes, and the research varies methodologically in both study duration and supplemented dose (8 to 96 weeks and 800 to 14,000 IU per day) [16,17,18,19,20,21,22,23,24,25,26,27,28]. The investigations do not provide a clear answer concerning the impact of supplementation on the immune system or the supplemented dose and serum VitD concentration necessary to exert beneficial effects. Furthermore, despite common VitD administration practices among MS patients, there are still no global recommendations indicating the need for supplementation and its principles.

The aim of this study was to determine whether VitD supplementation may affect immunological markers of inflammation in RRMS individuals and if the effect varies depending on the dose. Two different doses of oral VitD (2000 IU per day and 15,960 IU per month) were administered to 52 RRMS individuals. The levels of 25(OH)D, activated leukocyte adhesion molecule (ALCAM), CXC motif chemokine ligand 16 (CXCL16), pentraxin 3 (PTX3), interleukin-1 receptor antagonist (IL-1RA), and osteoprotegerin (OPG) were measured at the beginning of the study and after six months of VitD supplementation.

## 2. Results

A total of 52 RRMS individuals who fulfilled the inclusion and exclusion criteria were supplemented with VitD throughout the study and reported for a follow-up; the two study groups involved 23 patients (44.23%) who received a high dose (HD) and 29 (55.77%) patients who received a low dose (LD) of VitD.

Most patients were female (63.46%). The median age [years] was 39.5 (34.5–49.8) in the HD group and 47 (40.0–55.0) in the LD group. Significant differences were observed in age, body weight, and height between both study groups (*p* = 0.028, *p* = 0.014, and *p* = 0.001, respectively). The median body weight [kg] was significantly higher in the HD group (74.0 (65.0–89.0)) than in the LD group (65.0 (62.0–75.0)). There were no statistically important differences in BMI [kg/m^2^] (23.6 (22.8–27.1) in the HD group and 23.4 (22.1–26.8) in the LD group, *p* = 0.496). Obesity, defined as a BMI ≥ 25, was present in 34.78% of participants in the HD group and 31.03% of individuals in the LD group. Smoking cigarettes and regular physical activity were reported by 28.85% and 17.31% of patients, respectively. Table 1 shows the basic characteristics of the study participants.

The median initial 25(OH)D concentration [ng/mL] was 23.023 (15.578–25.76) in the HD group and 28.318 (20.644–32.232) in the LD group. We observed a significant increase after six months of VitD supplementation to 29.819 (24.937–38.064) and 30.837 (25.382–36.789), respectively (*p* < 0.01). The increase was significantly higher in the HD group (*p* = 0.01), where the 25(OH)D level was lower before the study and increased to a similar level in both groups at the follow-up. However, the dynamics of the increase were higher in the HD group (*p* = 0.01). The median serum level of 25(OH)D before and after VitD supplementation is given in Table 2.

**Figure 1:**
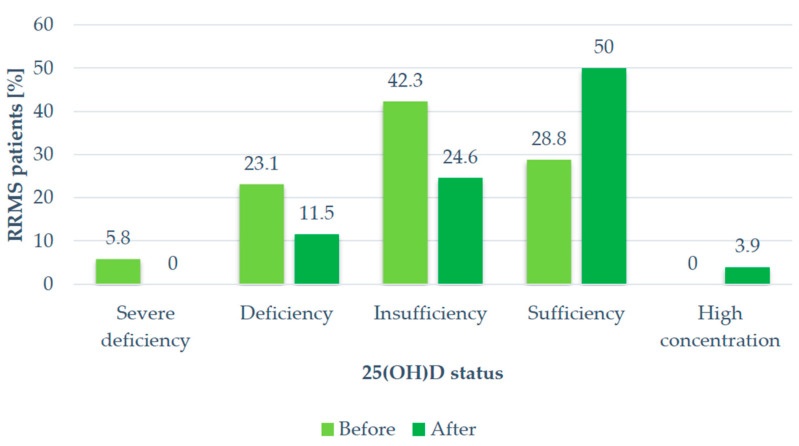
Vitamin D status of the study participants depending on 25(OH)D serum concentrations before and after vitamin D supplementation. RRMS: relapsing–remitting multiple sclerosis, 25(OH)D: 25-Hydroxyvitamin D.

Hypovitaminosis D, defined as a serum 25(OH)D level < 30 ng/mL, was found in most patients (71.2%). After supplementation, the serum VitD level was still insufficient in 46.2% of the participants. The VitD status of the study group depending on the 25(OH)D serum level at two different time points is given in Figure 1.

The median initial EDSS score in the study participants was 3.0 (2.5–3.5) and no significant changes were observed after VitD supplementation (*p* = 0.709). A significant increase in the concentrations of CXCL16, PTX3, and OPG was observed during the study (*p* = 0.02, *p* = 0.01, and *p* < 0.01, respectively). Furthermore, a significant dose-related association was found only in terms of PTX3 and OPG levels (*p* = 0.04 and *p* = 0.03, respectively). A higher increase in the above parameters was found in the LD group. We observed a decrease in IL-1RA levels in the HD group and an increase in the LD group, which was not statistically significant (*p* = 0.19). Additionally, the difference between the doses of VitD was not statistically significant (*p* = 0.32). Furthermore, a decrease in the concentrations of ALCAM was found during the study. However, it was not statistically significant (*p* = 0.87). The concentrations of selected immunological markers of inflammation before and after the administration of different doses of VitD are given in Table 2.

A significant positive correlation was found between the serum concentrations of 25(OH)D and PTX3 (R = 0.28, *p* < 0.05) and OPG (R = 0.28, *p* < 0.05) at the study onset. Correlations between the serum levels of VitD and the concentrations of the selected immunological markers of inflammation on the day of enrollment are given in Table 3.

## 3. Discussion

The significant differences in age, body weight, and height between the study groups might not have influenced the results of our study. The age difference between the groups did not affect VitD metabolism, as reported by some studies that found differences in metabolism in patients above 65 years of age [29]. According to the guidelines, higher doses of VitD should be supplemented in the elderly [30]. As regards body weight and height, the BMI levels showed no statistically significant differences, and due to the hydrophilic properties of VitD, it seems to be favorable to introduce higher doses in patients with higher amount of adipose tissue [31]. In addition, we did not observe any statistically significant differences in the initial levels of inflammatory markers between the HD group and the LD group.

Our study has demonstrated that hypovitaminosis D is frequent in MS patients (71.2%), which is in line with other reports [32]. At the end of the study, the 25(OH)D level was still below the recommended range in 46.2% of the participants, which may indicate insufficient VitD doses or insufficient duration of supplementation (especially in those with severe deficiency). Additionally, the baseline 25(OH)D level was lower in the HD group and the participants had higher body weight. It is caused by the fat solubility of VitD, which can be stored in adipose tissue. Furthermore, the participants had a more sedentary lifestyle, which was associated with lower sun exposure. The increase in VitD concentration was significant in both groups. However, the dynamics of the increase were higher in the HD group, which could be attributed to a higher cumulative dose of the supplement.

The pathogenesis of MS is complex and is associated with various inflammatory pathways and different immunological changes of different molecules and factors. Rosjo et al. found that some markers could be related to the clinical activity of the disease (IL-1RA or PTX3), radiological activity (CXCL16 and OPG) [33], or are involved in the migration of T-cells and myelin repair (ALCAM) [27]. ALCAM, CXCL16, and PTX3 are known for their pro-inflammatory actions, while IL-1RA and OPG may be responsible for the opposite effects [34]. Furthermore, they can be involved in an acute (ALCAM, IL-1RA, OPG) or acute and repair (CXCL16, PTX3) phase of inflammation [34]. Therefore, these laboratory parameters could be used as novel biomarkers and potential predictors of MS activity due to their potential ability to reflect and predict MS activity.

Our results showed a significant increase in the concentrations of pro-inflammatory CXCL16. It is one of the markers crucial in the pathogenesis of MS through its ability to regulate the migration of different leukocytes and leukocyte adhesion to the vascular wall, thereby controlling inflammation and immune responses. These processes constitute a pathological background of inflammatory diseases such as MS. Depending on the presence of a specific amino acid sequence, CXC chemokines can be divided into ELR+ (CXCL1-3, CXCL5-8, CXCL17) or ELR- (CXCL4, CXCL9-14, and CXCL16) [35]. CXCL16 is present on the surface of antigen-presenting cells (including macrophages and astrocytes), protecting the CNS against excitotoxic damage associated with intense glutamate exposure and glucose and oxygen deficiency [33]. The expression of CXCL16 is increased in patients with MS compared to healthy individuals and reflects the disease activity [36]. Its level increases in macrophages and astrocytes in the lesions [33]. In their double-blind pilot study, Sotirchos et al. assessed the immunological effect of cholecalciferol supplementation. The intervention group consisted of MS patients who were either untreated or treated with a different disease-modifying therapy (DMT), such as IFN-β, glatiramer acetate, natalizumab, fingolimod, and abatacept. Compared to low-dose treatment (800 IU daily), they noticed a significant increase in the serum 25(OH)D level in the HD group (10,400 IU daily). There were no changes in the levels of 51 cytokines and different chemokines (CXCL1, CXCL5, CXCL9, and CXCL10) in both study groups [17]. However, other researchers observed that serum CXCL16 levels were negatively correlated with the 25(OH)D level in patients with type 2 diabetes mellitus (DM2) [37]. It is confirmed that exposure to pro-inflammatory factors results in a higher expression of CXCL16 [33,38,39], which might explain the increase in this marker in our study. The increase in pro-inflammatory cytokines constitutes a pathogenetic basis of chronic inflammatory diseases such as MS and it is known that the disease progression correlates with abnormal cytokine expression [40]. Advancing age and chronic clinical conditions augment inflammation, which is known as inflammaging. Such a background also constitutes an important risk factor for infections [41]. Recent studies on COVID-19 patients have demonstrated that the expression of CXCL16 is upregulated during the inflammatory response and its level is higher in severe disease compared to healthy controls and individuals with a mild infection [41]. In our study, the increase in CXCL16 levels might be associated with different clinical conditions and radiological progression of the disease, which was not assessed in our study. According to Holmoy et al., increasing concentrations of CXCL16 are connected with lower odds ratios (ORs) for new T1Gd+ lesions and combined unique activity (CUA; the sum of T1Gd+ lesions and new or enlarging T2 lesions) [33]. It should be noted that clinically silent lesions on MRI are 5–10 times more frequently observed than reported clinical relapses [42].

Additionally, the levels of pro-inflammatory PTX3 and anti-inflammatory OPG significantly increased after six months of VitD supplementation and their increase was higher in the LD group. PTX3 is an acute-phase protein secreted locally after exposure to inflammatory factors, such as interleukin-1β (IL-1β) or tumor necrosis factor-α (TNF-α) [3,43]. It is released by different cell types, including neutrophils, macrophages, microglia, dendritic cells, fibroblasts, and endothelial cells. Apart from antibody-like activity, PTX3 plays a part in regulating inflammatory pathways [44]. MS is associated with higher plasma PTX3 levels [44], and during relapses, PTX3 levels are significantly elevated and correlate with the EDSS score. However, PTX3 levels are significantly lower during remission, with no association with EDSS scores [3]. In contrast to our study, Signoriello et al. found that therapy with glatiramer acetate was associated with reduced PTX3 levels [44]. However, Nowak-Kiczmer et al. indicated that serum PTX3 levels were correlated with the duration of MS [45], which might explain our findings. Furthermore, PTX3 may reflect a favorable outcome to some extent. It is suggested that in the late stages of inflammation, when IL-10 enhances the production of PTX3, it may restrict phagocytosis of damaged neurons and decrease migration of immune cells into inflamed regions [34]. A study on patients with rheumatoid arthritis found no correlations between PTX3 and biochemical measurements such as VitD [46]. In another study on patients with coronary artery disease, two-fold higher gene expression of PTX3 was noticed in a 25(OH)D-deficient group compared to patients with a normal range [47]. At the onset of our study, most patients were VitD-deficient and a significant correlation was found between serum concentrations of 25(OH)D and PTX3. Due to the participation of PTX3 in the inflammatory response, it has a crucial role in infectious diseases (their occurrence and progression) and different clinical conditions [3,43]. However, its increase in our study may be attributed to diverse concomitant factors, as in the case of CXCL16.

OPG is a member of the TNF receptor family and one of the markers associated with the pathogenesis of MS through regulating the binding between the receptor activator of nuclear factor-kB (RANK) and its ligand (RANKL). The axis is involved in the polarization and differentiation of macrophages, activation and recruitment of T-cells, and the dysregulation of the axis is related to the risk of autoimmune diseases, including MS, by association with T-cell tolerance loss and neural tissue damage [48]. Additionally, the RANK/RANKL/OPG axis is crucial for normal bone development and regulation of differentiation of osteoclasts and bone resorption [49]. RANKL may promote the differentiation of osteoclastic precursors and the activation of osteoclasts, whereas OPG inhibits the activity of RANKL [50]. Additionally, the axis is involved in different activities associated with injury and repair in the CNS through different immune interactions [48]. In inflammatory or autoimmune diseases, such as MS, RANKL expression might be induced through the activity of T-cells and different pro-inflammatory cytokines triggering bone loss [51]. The levels of OPG may also be modified by different cytokines [49], which might have affected our results. The increase in OPG levels might be related to elevated serum levels of PTX3 based on the reports, according to which PTX3 may intensify osteolytic changes in bones by stimulating osteoclastogenesis through increasing the osteoblast RANKL production [52]. At the onset of MS, the levels of OPG in the cerebrospinal fluid (CSF) are decreased, while serum levels of OPG are comparable in RRMS patients, subjects with the initial stage of MS, and healthy controls [53]. The gene expression of RANKL/RANK/OPG in peripheral blood mononuclear cells is higher only in RRMS [53]. Furthermore, higher concentrations of OPG [33] and RANKL are present in MS patients with low mean EDSS scores compared to the age-matched controls [51]. Therefore, in our study, the changes in OPG levels may have a pathological background related to MS and osteoporosis. MS is one of the factors contributing to long-term immobilization and a risk factor for low bone density [54]. VitD supplementation has a favorable influence on bone metabolism [55]. Other authors found that VitD was one of the endogenous factors affecting the soluble RANKL/OPG ratio in favor of the expression of OPG [48], which is in line with our study, which showed a positive correlation between VitD and OPG levels. As was already mentioned, the level of OPG may be associated with radiological disease activity because increasing concentrations of OPG are associated with lower OR for new T1Gd+ and T2 lesions and CUA, as reported by Holmoy et al. [33]. Additionally, in our study, the increase in OPG levels was higher in the LD group, which might be related to the significantly lower body weight of the participants compared to the HD group. Recent studies have demonstrated that overweight and obese MS patients had higher MRI activity during IFN-β therapy compared to patients with normal weight, which indicates that the BMI level may affect the response to treatment. However, some studies also concluded that the levels of PTX3 were inversely correlated with the BMI and IL-1RA was positively correlated with this index, which was not confirmed in our study (the increase in PTX3 levels was higher in the LD group and we did not observe any significant differences in the change in IL-1RA concentrations between the study groups) [56]. Furthermore, studies showed that intramuscular administration of IFN-β induced different time-dependent changes in OPG levels, affecting bone homeostasis in MS patients (the levels of OPG protein decreased 25% at the 8 h time point, then increased 43% at the 24 h time point) [54].

Furthermore, Rosjo et al. evaluated the influence of VitD supplementation (20,000 IU/week) in 68 RRMS patients on the same markers of inflammation and some additional factors, including transforming growth factor β (TGF-β), chemokine ligand 21 (CCL21), metalloproteinase-9 (MMP-9), osteopontin (OPN), soluble frizzled-related protein 3 (sFRP3), and tumor necrosis factor receptor 1 (TNF-r1). They observed a significant increase in serum 25(OH)D concentrations, which were higher than those in our study, with no influence on inflammatory parameters [27]. The differences in our results might be associated with a significantly longer duration of the study of Rosjo et al. and higher supplemental doses of VitD (the initial 25(OH)D concentrations were similar to our HD group). After completing the supplementation, the VitD levels were more than doubled in the study of Rosjo et al. (the mean level increased by approximately 27 ng/mL). In another study, Rosjo et al. found that IFN-β treatment of patients with well-established disease was associated with a slight increase in VitD serum levels but neither radiological nor biochemical effects related to IFN-β treatment were influenced by VitD status. Patients were categorized into patient quartiles by ascending individual seasonally adjusted mean 25(OH)D levels from baseline to month 3 (the mean level of 25(OH)D in the first patient quartile was 53.2, in the second, 66.0, in the third, 76.9, and in the fourth, 99.7 nmol/L). They observed a significant increase in mean levels of CXCL16, IL-1RA, and OPG across quartiles and of PTX3 in the first quartile after implementing therapy with IFN-β. The levels of the markers increased in each quartile after at least six months of treatment, which is in line with our study [57]. Other researchers also assessed the role of VitD in reducing different inflammatory agents, including interleukins, IFN-γ, TNF-α, TGF-β, colony-stimulating factors, and matrix metalloproteinases (MMPs). However, they found significant changes only in some inflammatory markers after VitD supplementation in MS patients [17,18,24,25,26,28].

Considering the role of VitD in reducing inflammation, the possible modulating effect of DMT should also be considered. Such therapy prevents relapses and the progression of disability by shifting immune responses from a pro-inflammatory toward an anti-inflammatory status [58]. According to Rosjo et al., the concentrations of CXCL16 and IL-1RA were significantly higher in patients on DMT compared to untreated individuals. No synergistic effects of DMT and VitD supplementation were found on inflammatory markers. However, a more anti-inflammatory phenotype was observed in patients undergoing treatment [27]. Our study found no clear associations between the type of immunomodulatory treatment and its influence on inflammation. However, the correlation could be more visible in larger cohorts, including patients with untreated MS.

To conclude, significant changes in the concentrations of some inflammatory markers might reflect the clinical and radiological activity of MS [27] and could indicate a favorable outcome considering MRI findings. None of the participants reported a relapse during the study and the median EDSS level was the same at the follow-up. However, undiagnosed relapses might have been confused with infectious symptoms [59] occurring more frequently in the winter–autumn period. They might have been related to the COVID-19 pandemic. Recent studies have indicated that isolated relapses without changes in the EDSS score may also occur [42]. Additionally, most of the assessed parameters may be induced by the action of pro-inflammatory cytokines, which cannot exclude the participation of factors other than MS. Significant changes in several markers might also be explained by other confounding clinical conditions that could affect their levels. Furthermore, the immunological parameters play an essential role in inflammation, but they can also mediate neuroprotection and repair [33] during MS [60] and are also induced by immunomodulatory treatment, which may explain different changes in the study period. In addition, significant correlations between the serum levels of 25(OH)D and different inflammatory markers observed only at the beginning of the study indicate that VitD could have an anti-inflammatory action in MS. However, this may be more pronounced during VitD deficiency. This finding is in line with other reports which found that the action of VitD could be more pronounced in patients who are VitD-deficient (<50 nmol/L), causing more visible immunological changes after supplementation [61]. Generally, VitD may have a favorable influence on some inflammatory markers.

In our study, the doses of VitD could be too low to induce an anti-inflammatory effect. Therefore, all the above processes could contribute to the observed changes in the concentrations of selected markers of inflammation.

## 4. Material and Methods

### 4.1. Study Design and Patient Characteristics

Adult patients treated in the Multiple Sclerosis Center in Zabrze, Poland, with MS diagnosed based on the revised McDonald criteria (2017) [62], were included in the study. The inclusion criteria were immunomodulatory treatment for at least six months and residence in the region of Silesia (latitude 49–50° N). The exclusion criteria were as follows: neurological diseases other than MS, disorders affecting calcium-phosphate homeostasis, phenotypes of MS other than RRMS, a history of relapse in the six months before the inclusion, current Expanded Disability Status Scale (EDSS) score > 5, use of drugs or a diet that could influence calcium-phosphate metabolism (including glucocorticosteroids) within six months before the inclusion, VitD supplementation within six months before the study, traveling to different climatic zones in the last six months, staying indoors only or working underground, menopausal period, current or planned pregnancy within the next six months, and breastfeeding. None of the participants changed, discontinued DMT, or reported a relapse during the study.

Out of 309 patients, we selected 52 subjects who met the inclusion and exclusion criteria, gave informed consent to participate in the study, and reported for the follow-up at two time points. The study group was divided into two subgroups. Different regimens of 6-month VitD supplementation were implemented:

Low-dose (LD) group: 29 MS patients with a low dose of VitD (calcifediol: 15,960 IU/month, i.e., 530 IU/day);

High-dose (HD) group: 23 MS patients with a high dose of VitD (cholecalciferol: 2000 IU/day).

All participants were on DMT, including interferon beta-1b (IFNβ-1b) (*n* = 5), IFNβ-1a (*n* = 5), pegylated IFNβ-1a (*n* = 3), dimethyl fumarate (*n*= 21), teriflunomide (*n* = 8), ocrelizumab (*n* = 6), fingolimod (*n* = 2), and cladribine (*n* = 2).

### 4.2. Serum Measurements

Blood samples were obtained from each patient on the day of inclusion from October 2021 to March 2022 (baseline) and after six months of VitD supplementation. The serum samples were frozen and kept at −80 °C until further analysis. All analyses were performed at the Department of Biochemistry, Faculty of Medical Sciences in Zabrze, Medical University of Silesia, Katowice, Poland.

Serum concentrations of VitD [25(OH)D] were determined using a sandwich enzyme-linked immunosorbent assay (ELISA) kit (IDK^®^ 25-OH-Vitamin D ELISA, Immuniq, Żory, Poland). The 25(OH)D status was specified depending on the serum level: VitD sufficiency (≥30.0 ng/mL), VitD insufficiency (20.0–29.9 ng/mL), VitD deficiency (<20.0 ng/mL), and severe deficiency (<10.0 ng/mL) [63]. Hypovitaminosis was considered when a serum concentration of 25(OH)D < 30.0 ng/mL [64,65].

For the quantitative determinations of ALCAM, CXCL16, PTX3, Il-1RA, and OPG levels, the ELISA kits were used according to the manufacturer’s recommendations (Immuniq, Żory, Poland). The analysis of each parameter was performed separately. Specific antibodies were pre-coated onto a microplate. Standards and samples were added to the wells and a specific antigen was bound by the immobilized antibody. After washing out the unbound substances, a specific biotin-conjugated antibody was added into the wells and washed again. Next, avidin conjugated to horseradish peroxidase (HRP) was introduced to the wells and washed again. Adding a substrate solution resulted in a change in color proportional to the amount of each parameter bound in the initial step, which was measured spectrophotometrically at a wavelength of 450 nm after adding the stopping solution. The concentrations of the above markers in the samples were read from the standard curves.

### 4.3. Statistical Analysis

All data not normally distributed are presented as median and interquartile range. The Wilcoxon test for dependent measurements was performed to compare quantitative variables. The Spearman’s Rho coefficient was used to estimate correlations between the parameters. The analysis was conducted in the RStudio (Posit, Boston, MA) environment using the R language.

The relationship between the dose of VitD supplementation and changes in the immunological markers over time was determined based on the nparLD test, which was used to calculate *p*-values for the time effect, group effect, and the interaction between the group effect and time. The results were considered statistically significant at *p* ≤ 0.05.

The GEE model with adjustment for the BMI and age was performed to confirm the obtained results (Supplementary Material).

## 5. Conclusions

There is emerging evidence that VitD may be associated with immunological benefits, being responsible for controlling inflammation. However, there is no consensus regarding the dose and duration of supplementation.

We showed that in patients with RRMS who were given different DMT, the doses of VitD in the study groups might be too low to induce beneficial effects on inflammation. Low-dose VitD supplementation, depending on the initial 25(OH)D concentration, may be sufficient to normalize serum VitD levels. However, the dose needed to induce immunological effects may be different. Nevertheless, we observed significant changes in several immunological markers of inflammation. We found a significant increase in the pro-inflammatory actions of CXCL16 and PTX3. We also found a statistically significant decrease in the OPG levels in the follow-up. It is still uncertain why the markers showed different changes. Therefore, the influence of DMT and factors other than VitD supplementation should be considered. There were some significant differences in the results depending on the supplemented dose of VitD. However, these associations are difficult to explain.

Considering the favorable impact of VitD, MS patients may benefit from VitD supplementation. The question of whether it improves inflammation is still open. Low doses of VitD may be insufficient to produce positive effects, especially in terms of reduction in inflammation in MS patients. Further research is warranted to determine the role of 25(OH)D in MS immunopathology and a dose that could reduce the disease activity.

Effective treatment of MS should target the mechanisms underlying the disease. However, considering the participation of the assessed markers in the pathogenesis of MS, further studies should be related to targeting these particles. 

## 6. Limitations

The study has some limitations: a small number of participants and no control or placebo group. Therefore, our results should be treated as preliminary.

Radiological images of patients were not assessed because MRI examinations were planned in different stages of the study, as determined in the drug program. However, it would be useful to assess radiological activity of the disease and correlations with the levels of the inflammatory markers.

It is difficult to exclude the influence of other individual and environmental factors on inflammation. Therefore, it may be favorable to detect changes in these markers in the CSF with the assessment of basic inflammatory parameters in serum. Nutritional status and gene polymorphisms of the study participants should also be considered.

## Figures and Tables

**Table 1:** The characteristics of the study group based on VitD supplementation.

	HD ^1^ Group			LD ^2^ Group			*p*
Variable	Median	q1	q3	Median	q1	q3	
Age [years]	39.5	34.5	49.8	47.0	40.0	55.0	0.028
Weight [kg]	74.0	65.0	89.0	65.0	62.0	75.0	0.014
Height [m]	1.8	1.7	1.8	1.7	1.6	1.8	0.001
BMI [kg/m^2^]	23.6	22.8	27.1	23.4	22.1	26.8	0.496
Age at diagnosis of MS ^3^ [years]	34.0	26.0	47.0	33.5	28.5	43.8	0.626
Disease duration [years]	6.0	4.0	12.0	8.0	3.0	15.8	0.215
Age at first symptoms [years]	31.0	24.5	43.0	32.0	27.5	39.3	0.601
Duration of immunomodulatory treatment [months]	53.0	42.0	104.0	80.5	41.3	132.8	0.866
The number of MS relapses	2.0	1.0	4.0	3.0	1.0	5.3	0.240
Time from the last relapse [months]	56.0	31.5	70.0	55.0	33.5	82.0	0.724
The number of relapses treated with GCs ^3^	2.0	1.0	3.5	2.0	1.0	5.5	0.125
Time from the last administration of GCs [months]	58.0	37.0	77.0	55.0	31.5	74.5	0.652
EDSS ^4^ [score]	3.0	2.5	3.5	3.0	2.5	3.5	0.746
Time from smoking initiation [years]	20.0	10.0	27.5	20.0	18.8	27.5	0.396
Smoking [cigarettes/day]	15.0	11.3	18.8	12.5	10.0	18.8	0.622
Physical activity [min/week]	165.0	67.5	292.5	120.0	60.0	210.0	0.368

^1^ High dose of supplemental vitamin D, ^2^ low dose of supplemental vitamin D, ^3^ multiple sclerosis, ^3^ glucocorticosteroids, ^4^ Expanded Disability Status Scale.

**Table 2:** Serum concentrations of 25(OH)D and selected oxidative stress markers before and after vitamin D supplementation.

		Before			After			GE ^7^ (*p*)	TE ^8^ (*p*)	GETE ^9^ (*p*)
		Median	q1	q3	Median	q1	q3			
25(OH)D ^1^ [ng/mL]	HD	23.023	15.578	25.76	29.819	24.937	38.064	0.19	0.00	0.01
	LD	28.318	20.644	32.232	30.837	25.382	36.789			
ALCAM ^2^ [ng/mL]	HD	2.205	1.469	3.044	1.937	1.51	3.035	0.84	0.87	0.86
	LD	2.071	1.335	3.124	2.014	1.437	2.669			
CXCL16 ^3^ [ng/mL]	HD	0.16	0	0.624	0.232	0.113	0.471	0.62	0.02	0.11
	LD	0.239	0	0.526	0.407	0.21	0.717			
PTX3 ^4^ [pg/mL]	HD	2334.951	1612.287	2772.34	2377.47	1872.12	2861.41	0.95	0.01	0.04
	LD	2095.914	1631.604	2725.131	2378.898	2092.791	3123.678			
IL-1Ra ^5^ [pg/mL]	HD	1300.264	781.511	2003.067	937.74	621.433	1671.224	0.60	0.19	0.32
	LD	1030.524	921.694	1872.212	1132.398	834.04	1665.806			
OPG ^6^ [pmol/L]	HD	5.451	4.301	6.448	6.323	5.825	6.931	0.26	0.00	0.03
	LD	5.304	4.733	6.085	7.065	6.289	7.454			

^1^ 25-Hydroxyvitamin D, ^2^ activated leukocyte adhesion molecule, ^3^ CXC motif chemokine ligand 16, ^4^ pentraxin 3, ^5^ interleukin-1 receptor antagonist, ^6^ osteoprotegerin, ^7^ group effect, ^8^ time effect, ^9^ group effect × time effect.

**Table 3:** Correlations between vitamin D serum levels and the concentrations of selected immunological markers of inflammation before VitD supplementation.

Time	Variable 1	Variable 2	R	*p*
Before	25(OH)D ^1^	PTX3 ^2^	0.28	0.0479
		OPG ^3^	0.28	0.0457

^1^ 25-Hydroxyvitamin D, ^2^ PTX3: pentraxin 3, ^3^ OPG: osteoprotegerin.

## Data Availability

The dataset obtained in the research is available from the corresponding author on reasonable request.

## Supplementary Materials

The following supporting information can be downloaded at: https://www.mdpi.com/article/10.3390/ijms25147592/s1.
