# Supplementary material for: Can Vitamin D Reduce Inflammation? The Influence of Supplementation on Selected Immunological Markers
Source: Int J Mol Sci. 2024 Jul 11;25(14):7592. doi: 10.3390/ijms25147592 (PMC11277077; doi:10.3390/ijms25147592)
Supplement: Supplementary file 1 [file ijms-25-07592-s001.zip › ijms-3043856-supplementary.pdf]

```
Call:
geeglm(formula = `25OHD` ~ Time + Supplemented_dose +
    BMI, data = ., id = Lp)

 Coefficients:
                  Estimate Std.err  Wald Pr(>|W|)
(Intercept)         15.414   3.541 18.95  1.3e-05 ***
Time                 6.635   1.788 13.78  0.00021 ***
Supplemented_dose    3.030   1.783  2.89  0.08915 .
BMI                  0.031   0.103  0.09  0.76279
---
Signif. codes:  0 '***' 0.001 '**' 0.01 '*' 0.05 '.' 0.1 ' ' 1

Correlation structure = independence
Estimated Scale Parameters:

            Estimate Std.err
(Intercept)     83.1    12.6
Number of clusters:   104  Maximum cluster size: 1


Call:
geeglm(formula = ALCAM ~ Time + Supplemented_dose + BMI, data = .,
    id = Lp)

 Coefficients:
                  Estimate  Std.err  Wald Pr(>|W|)
(Intercept)        2.77463  0.74744 13.78  0.00021 ***
Time              -0.22662  0.30850  0.54  0.46260
Supplemented_dose -0.08786  0.30970  0.08  0.77666
BMI                0.00388  0.01825  0.05  0.83168
```

```
---
Signif. codes:  0 ‘***’ 0.001 ‘**’ 0.01 ‘*’ 0.05 ‘.’ 0.1 ‘ ’ 1

Correlation structure = independence
Estimated Scale Parameters:

            Estimate Std.err
(Intercept)     2.47   0.546
Number of clusters:   104  Maximum cluster size: 1


Call:
geeglm(formula = CXCL16 ~ Time + Supplemented_dose + BMI, data = .,
    id = Lp)

 Coefficients:
                  Estimate Std.err Wald Pr(>|W|)
(Intercept)        0.00340 0.21229 0.00    0.987
Time               0.11171 0.08458 1.74    0.187
Supplemented_dose  0.01637 0.08505 0.04    0.847
BMI                0.00837 0.00472 3.14    0.076 .
---
Signif. codes:  0 ‘***’ 0.001 ‘**’ 0.01 ‘*’ 0.05 ‘.’ 0.1 ‘ ’ 1

Correlation structure = independence
Estimated Scale Parameters:

            Estimate Std.err
(Intercept)    0.186   0.049
Number of clusters:   104  Maximum cluster size: 1
```

```
Call:
geeglm(formula = PTX_3 ~ Time + Suplementowal_dawke + BMI, data = .,
   id = Lp)

 Coefficients:
                 Estimate Std.err  Wald Pr(>|W|)
(Intercept)          2448.5   385.3 40.39  2.1e-10 ***
Time                 271.9   139.0  3.83    0.050 .
Supplemented_dose      14.9   141.0  0.01    0.916
BMI                  -19.0    10.9  3.02    0.082 .
---
Signif. codes:  0 '***' 0.001 '**' 0.01 '*' 0.05 '.' 0.1 ' ' 1

Correlation structure = independence
Estimated Scale Parameters:

          Estimate Std.err
(Intercept)   502143   60314
Number of clusters:   104  Maximum cluster size: 1


Call:
geeglm(formula = IL_1RA ~ Time + Suplementowal_dawke + BMI, data = .,
   id = Lp)

 Coefficients:
                 Estimate Std.err Wald Pr(>|W|)
(Intercept)          1145.3   556.1 4.24    0.039 *
Time                 -45.6   184.9 0.06    0.805
Supplemented_dose      42.1   181.7 0.05    0.817
```

```
BMI                   12.4    17.5 0.50    0.479
---
Signif. codes:  0 '***' 0.001 '**' 0.01 '*' 0.05 '.' 0.1 ' ' 1

Correlation structure = independence
Estimated Scale Parameters:

            Estimate Std.err
(Intercept)   888867  198728
Number of clusters:   104  Maximum cluster size: 1



Call:
geeglm(formula = OPG ~ Time + Supplemented_dose + BMI, data = .,
    id = Lp)

 Coefficients:
                   Estimate Std.err  Wald Pr(>|W|)
(Intercept)          3.4967  0.7406 22.29  2.3e-06 ***
Time                 1.2036  0.2796 18.53  1.7e-05 ***
Supplemented_dose    0.4184  0.2815  2.21     0.14
BMI                  0.0266  0.0205  1.69     0.19
---
Signif. codes:  0 '***' 0.001 '**' 0.01 '*' 0.05 '.' 0.1 ' ' 1

Correlation structure = independence
Estimated Scale Parameters:

            Estimate Std.err
(Intercept)     2.03   0.379
Number of clusters:   104  Maximum cluster size: 1
```

```
Call:
geeglm(formula = `25OHD` ~ Time + Supplemented_dose +
    Age_years, data = ., id = Lp)

 Coefficients:
                  Estimate Std.err  Wald Pr(>|W|)
(Intercept)        11.7035  4.6411  6.36  0.01168 *
Time                6.6119  1.7872 13.69  0.00022 ***
Supplemented_dose   3.0862  1.7644  3.06  0.08026 .
Age_years           0.0950  0.0851  1.25  0.26383
---
Signif. codes:  0 ‘***’ 0.001 ‘**’ 0.01 ‘*’ 0.05 ‘.’ 0.1 ‘ ’ 1

Correlation structure = independence
Estimated Scale Parameters:

            Estimate Std.err
(Intercept)     81.4    12.8
Number of clusters:   102  Maximum cluster size: 1


Call:
geeglm(formula = ALCAM ~ Time + Supplemented_dose + Age_years,
    data = ., id = Lp)

 Coefficients:
                  Estimate Std.err Wald Pr(>|W|)
(Intercept)         1.6844  0.6758 6.21    0.013 *
Time               -0.2757  0.3056 0.81    0.367
```

```
Suplemented_dose     -0.1818  0.3256 0.31    0.577
Age_years            0.0287  0.0167 2.96    0.085 .
---
Signif. codes:  0 ‘***’ 0.001 ‘**’ 0.01 ‘*’ 0.05 ‘.’ 0.1 ‘ ’ 1

Correlation structure = independence
Estimated Scale Parameters:

            Estimate Std.err
(Intercept)     2.38   0.476
Number of clusters:   102  Maximum cluster size: 1


Call:
geeglm(formula = CXCL16 ~ Time + Suplemented_dose + Age_years,
    data = ., id = Lp)

 Coefficients:
                 Estimate  Std.err Wald Pr(>|W|)
(Intercept)        0.000109 0.247074 0.00     1.00
Time               0.123824 0.084037 2.17     0.14
Suplemented_dose    0.035615 0.080134 0.20     0.66
Age_years          0.003884 0.003294 1.39     0.24

Correlation structure = independence
Estimated Scale Parameters:

            Estimate Std.err
(Intercept)     0.18  0.0492
Number of clusters:   102  Maximum cluster size: 1
```

```
Call:
geeglm(formula = PTX_3 ~ Time + Suplementowal_dawke + Wiek_lata,
    data = ., id = Lp)

 Coefficients:
                 Estimate Std.err  Wald Pr(>|W|)
(Intercept)         2262.54  357.71 40.01  2.5e-10 ***
Time                291.86  140.45  4.32    0.038 *
Suplemented_dose       72.32  157.09  0.21    0.645
Age_years             -8.38    6.52  1.65    0.199
---
Signif. codes:  0 ‘***’ 0.001 ‘**’ 0.01 ‘*’ 0.05 ‘.’ 0.1 ‘ ’ 1

Correlation structure = independence
Estimated Scale Parameters:

           Estimate Std.err
(Intercept)   502992   68416
Number of clusters:   102  Maximum cluster size: 1
```

```
Call:
geeglm(formula = IL_1RA ~ Time + Suplemented_dose + Age_years,
    data = ., id = Lp)

 Coefficients:
                 Estimate Std.err  Wald Pr(>|W|)
(Intercept)         2467.03  426.30 33.49  7.2e-09 ***
Time                -44.04  176.23  0.06  0.80266
Suplemented_dose      238.82  183.31  1.70  0.19264
```

```
Age_years           -25.34    6.88 13.57  0.00023 ***
---
Signif. codes:  0 '***' 0.001 '**' 0.01 '*' 0.05 '.' 0.1 ' ' 1

Correlation structure = independence
Estimated Scale Parameters:

            Estimate Std.err
(Intercept)   791979  186132
Number of clusters:   102  Maximum cluster size: 1
```

```
Call:
geeglm(formula = OPG ~ Time + Suplemented_dose + age_years,
    data = ., id = Lp)

 Coefficients:
                 Estimate Std.err  Wald Pr(>|W|)
(Intercept)         2.3709  0.5827 16.56  4.7e-05 ***
Time                1.2150  0.2545 22.80  1.8e-06 ***
Suplemented_dose    0.4340  0.2508  2.99  0.08358 .
Age_years           0.0377  0.0098 14.83  0.00012 ***
---
Signif. codes:  0 '***' 0.001 '**' 0.01 '*' 0.05 '.' 0.1 ' ' 1

Correlation structure = independence
Estimated Scale Parameters:

            Estimate Std.err
(Intercept)     1.65    0.33
Number of clusters:   102  Maximum cluster size: 1
```
